# Zero-shot design of a *de novo* metalloenzyme

**DOI:** 10.64898/2026.04.23.720277

**Published:** 2026-04-24

**Authors:** Gina El Nesr, Simon L. Dürr, Irimpan I. Mathews, Qi Wen, Kewei Zhao, Ritimukta Sarangi, Ursula Röthlisberger, Fanny Sunden, Po-Ssu Huang

**Affiliations:** 1 Biophysics Program, Stanford University, Stanford, CA; 2 Institute of Chemical Sciences and Engineering, École Polytechnique Fédérale de Lausanne (EPFL), Lausanne, Switzerland; 3 Institute of Life Sciences, HES-SO Valais-Wallis, Sion, Switzerland; 4 Department of Bioengineering, Stanford University, Stanford, CA; 5 Stanford Synchrotron Radiation Lightsource SLAC National Laboratory, Menlo Park, CA; 6 Department of Biochemistry, Stanford University, Stanford, CA

## Abstract

The *de novo* design of enzymes remains a central challenge, requiring consideration of catalytic mechanism and optimization across biochemical and biophysical criteria. To capture these criteria, we draw on principles from evolutionary biology. Here, we present dEVA (design by EVolutionary Algorithm), a multi-objective design framework for structure-based protein design. We apply dEVA to the zero-shot, *de novo* design of metalloenzymes by optimizing for the coordination sphere of catalytic metals. We fully characterize one of these designs: a bi-zinc metalloenzyme exhibiting promiscuous hydrolytic activity towards both phosphomonoesters and phosphodiesters. This design achieves a catalytic efficiency (k_cat_/K_M_) of up to 1500 M^−1^s^−1^ and a rate enhancement ((k_cat_/K_M_)/k_w_) of up to 3 × 10^13^, comparable to characterized natural phosphatases. dEVA offers a general and modular strategy for the programmable design of protein function without dependence on natural templates, predefined motif, or evolutionary information.

The ability to design *de novo* proteins has been transformed by deep learning, from fragment-assembly methods to a plethora of generative models that vastly expanded the accessible design space^1^. Recent advances have extended these capabilities to multi-objective settings, particularly through sequence-structure co-design models that aim to jointly optimize sequence compatibility with structural fidelity^2^.

Current approaches for the design of functional proteins have borrowed active sites from nature. For enzymes, the residues directly facilitating catalysis (i.e., the catalytic motif) are extracted from natural enzyme structures and placed into designed structures. This methodology is intended to maintain the motif geometry and hopefully recapitulate catalytic function. However, optimizing for the structural motif alone does not guarantee catalytic competence. Rather, efficient catalysis requires other considerations; activity depends on a precise balance of chemical, geometric, and electrostatic criteria^3,4^.

Development of deep learning design methods capable of optimizing across multiple criteria abound. They rely on manual tuning of competing objective weights^5^, incorporating multiple filtering steps, or predicting protein structures^6,7^. The resulting designs often favor protein sequences that score well across individual criteria, but do not explicitly enforce compatibility between them. We take inspiration from recent methods to develop a framework that enables optimization of the physiochemical features necessary for efficient catalysis. We hypothesize that this will enable the design of *de novo* enzymes capable of catalyzing demanding reactions with comparable efficiencies to natural enzymes.

To address these challenges, we are motivated by how nature evolves highly proficient function: through iterative selective pressure. We propose dEVA (design by EVolutionary Algorithm), a generalizable protein design protocol for multi-objective, structure-based protein design. Based on the non-dominated sorting genetic algorithm (NSGA-II)^8^, dEVA allows for the explicit encoding of multiple design objectives—each defined as a function or model tailored to a desired property (Fig. 1A, Fig S1). Rather than optimizing for a single score, dEVA iteratively enriches for candidates that satisfy multiple design criteria simultaneously. In doing so, dEVA favors designs in which the specified objectives are mutually compatible and jointly probable.

In this framework, dEVA imposes no requirement for structure prediction confidence or predefined motifs to generate biophysically plausible designs, although these can be incorporated as additional objectives. dEVA operates zero-shot, iterating over generations without manual interference to refine the population of designs towards the Pareto front. Each sequence candidate is treated as an individual, with mutations and crossovers introduced as residue-level changes to diversify the explored sequence space. Optimal designs are identified at the knee point of the Pareto front: the sequence representing the best, balanced tradeoffs across all defined objectives (Fig 1B).

Here, we sought to apply dEVA to design a *de novo* metalloenzyme. In metalloenzymes, the catalytic motif is the first-shell residues directly coordinating the metal. However, the second-shell residues around the motif play an important role in mediating affinity, specificity, and reactivity^9^. Previous attempts at designing metalloproteins have leveraged either motif-scaffolding or rational design to achieve the desired metal binding^10–16^. A key attribute that has remained difficult is incorporating second-shell interactions into the designs^17^. Metalloenzymes impose an additional layer of complexity wherein the metal must occupy a catalytically competent coordination sphere to facilitate the chemical reaction, activate a nucleophile, and mediate electron transfer^18^. We therefore began by testing dEVA’s ability to design metalloproteins as a foundation for the more demanding challenge of designing metalloenzymes.

## The design of *de novo* metalloproteins.

**Figure 1.**
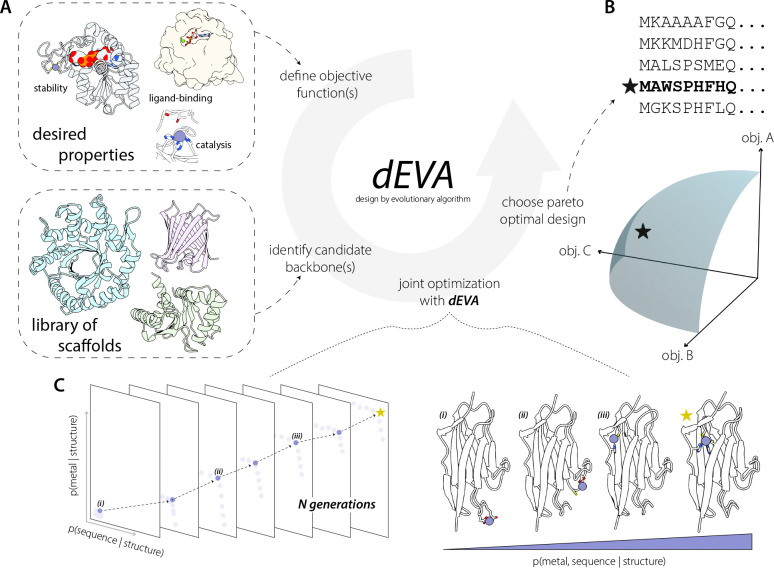
Overview of dEVA. (A) This multi-objective algorithm is a fixed-backbone, multi-objective optimization algorithm that can incorporate any number of defined objectives. (B) Optimal solutions are identified at the knee point (star) along the Pareto front. (C) dEVA follows a standard non-dominated genetic sorting algorithm (NSGA-II)^7^ where the initial population of sequences is generated, evaluated across multiple objectives, and iteratively refined through mutation and cross-over. The knee point represents the optimal tradeoff between sequence likelihood and metal-binding probability. Illustrated are representative designs sampled from along the Pareto front. Moving from *i* to *iii*, the predicted metal-binding geometry improves as the solutions approach the knee point, with *star* representing the optimal design at the knee point.

We began by posing the metalloprotein design as a fixed-backbone problem, where the goal is to identify sequences and metal configurations that are jointly co-adapted given a structural scaffold. This is achieved by defining two objectives in dEVA: *p(sequence | metal, structure)* and *p(metal | sequence, structure)*. For the former sequence objective, we use LigandMPNN^19^, trained to consider non-protein atoms. For the latter metal site design objective, we use Metal3D^20^ to predict metal location from the local chemical environment. At each iteration of the dEVA design protocol, LigandMPNN proposes mutations and model sidechains followed by Metal3D predicting the location of the metal ion(s). After *N* iterations, the final population reaches the Pareto front (Fig. 1C, Fig. S2).

We generated a diverse library of non-all-helical scaffolds using Protpardelle^21,22^ and applied dEVA to unconditionally design metal-binding sites. From the initial designs, we selected eight candidates spanning a range of backbone lengths, protein folds, structure prediction confidences, and coordination motifs for in depth experimental characterization (see **Methods,** Fig. S3–S5, Table S1–2), where the full sequence and proposed metal-binding site was designed entirely by dEVA. All eight proteins were well-expressed in *E. coli* and purified as soluble monomers confirmed by SDS-PAGE and size exclusion chromatography (SEC) (Fig. S6). Comprehensive characterization of all eight designs—including binding affinities, alanine point mutants, circular dichroism (CD) spectra in apo and holo states, and inductively coupled mass spectrometry (ICP-MS) data—is reported in full in Supplementary Materials (Figs S7–S9, Table S3).

**Figure 2.**
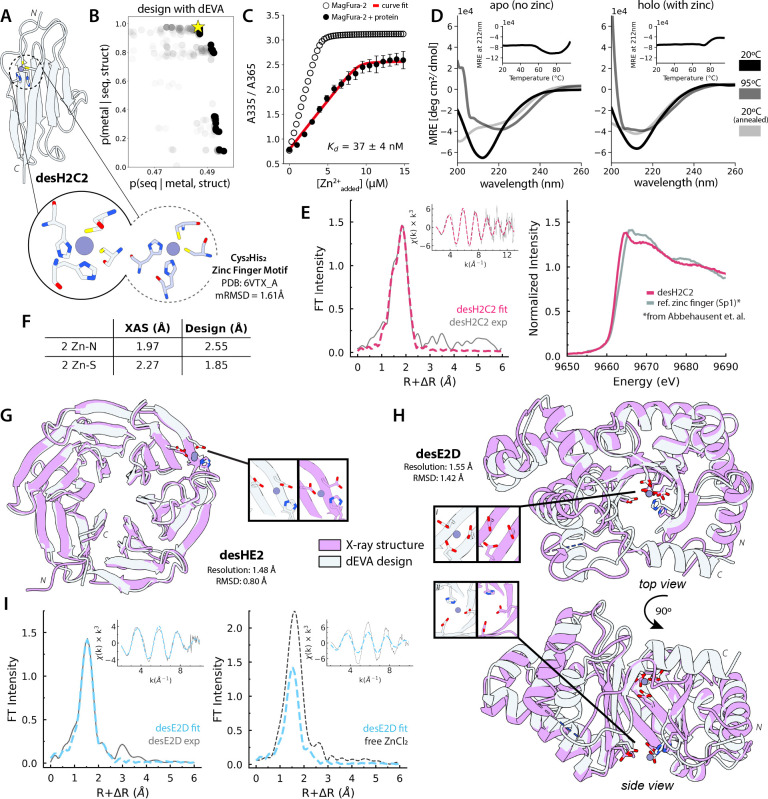
Designing de novo metalloproteins. (A) Cartoon representation of the designed metalloprotein desH2C2, where the gray circle represents the modeled zinc. The blow-out compares the designed motif (left) and its nearest neighbor motif in the PDB (right). (B) The yellow star indicates the chosen design from dEVA, which is also the Pareto-optimal solution sitting along the Pareto front. (C) The protein binds zinc tightly (K_d_=37±4nM) determined by competition titration against MagFura-2. Open circular dots represent experimental curve of MagFura-2 alone, black dots represent average experimental value, and red line represents best competition binding curve fit. Error bars represent relative error across n = 5 replicates. (D) Circular dichroism spectra with inset are thermal denaturation curves. The design is unstable in the apo form but shows increased thermostability in the presence of zinc. (E) X-ray absorption spectroscopy (XAS) of desH2C2 confirms metal coordination geometry. Comparison of the normalized Zn K-edge spectrum of desH2C2 (pink) with a digitized canonical zinc finger motif^23^ (grey) suggests the design is a *de novo* binding motif. (F) The fits are consistent with 2 histidines and 2 cysteines and closely match the design. (G) Crystal structure of (magenta) overlaid with design model (white) for desHE2 (1.48Å resolution, RMSD 0.80Å) and (H) desE2D (1.55Å resolution, RMSD 1.42Å). (I) Zn K-edge XAS spectra of desE2D confirms that zinc is bound to the protein ligands despite not being visible in the crystal structure.

**Figure 3.**
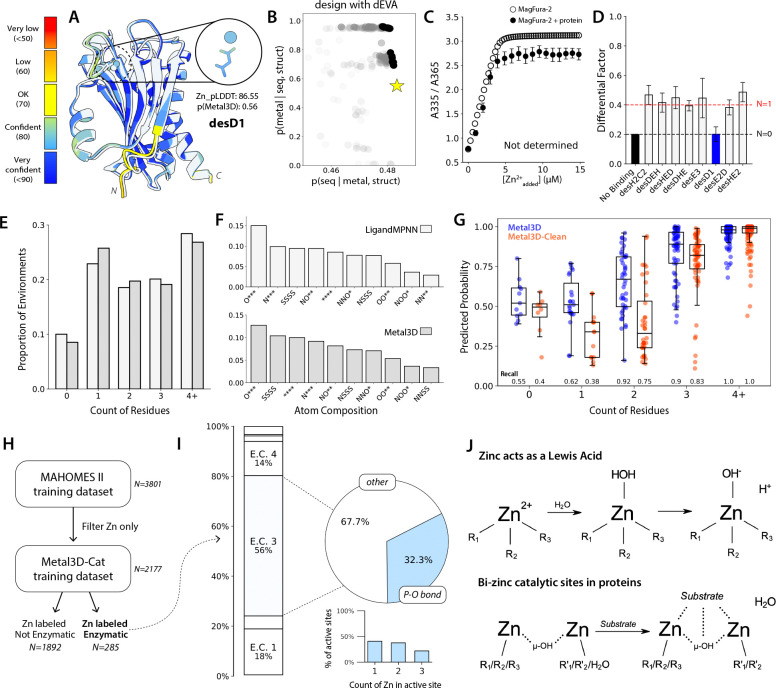
dEVA is sensitive to its models’ underlying training data. (A) AlphaFold-3 assigns high confidence to single ligand-coordinated zinc as indicated by desD1. Model in white overlaid with the predicted model, colored by pLDDT. (B) dEVA assigns the designed structure as a low-probability metal binding site. (C) The design has no detectable binding by MagFura-2 competition titration and (D) no detectable binding in ICP-MS. Error bars represent relative error for n=5 and n=3 replicates, respectively. (E) >54% of protein structures in the PDB have two or less coordinating ligands within 3.0Å of the zinc. (F) The majority of these structures have a zinc coordinated by a single carboxylate. The star (*) represents an open coordination sphere. (G) Retraining Metal3D (blue) by removing all metals coordinated with two or fewer ligands led to Metal3D-Clean (orange), reducing recall for under-coordinated metals while retaining recall for well-coordinated sites. Points represent all data with predicted probability greater than 0.0; boxes indicate IQR with median; whiskers extend to 1.5xIQR. (H) Curation of an enzymatic zinc training dataset. (I) Zinc sites span enzymatic classes, with the majority exhibiting hydrolytic behavior, including hydrolysis of a phosphate-oxygen bond. (J) Zinc act as a Lewis acid, and bi-catalytic sites can coordinate and facilitate hydrolysis.

Among all the designs, our best is desH2C2, an immunoglobulin-like fold in which dEVA designed a His_2_Cys_2_ coordination site without a motif template (Fig. 2A). desH2C2 binds zinc tightly (K_d_ = 37 ± 4 nM, Fig. 2B) and ICP-MS confirms 1:1 stoichiometry (Table S3, Fig 3D). Zinc confers thermal stability absent in the apo form by CD (Fig. 2C), consistent with natural metal-dependent stabilization. To resolve the precise local metal environment, we turned to X-ray absorption spectroscopy (XAS), a powerful solution-state structural technique that precisely characterizes the metal coordination shell. For desH2C2, the Zn K-edge X-ray absorption fine structure (EXAFS) was best fit to 2 Zn-N and 2 Zn-S, establishing coordination number and distances (Fig. 2D, Table S4). The normalized Zn K-edge spectrum reveals subtle whiteline differences compared to canonical zinc fingers^23^ (Fig. 2E), supporting the design’s distinct structural context and rotamer orientations. Together, these results demonstrate that dEVA has produced a *de novo* coordination geometry unique from structurally characterized zinc-containing proteins in the Protein Data Bank (PDB).

Crystal structures of three other designs validate that dEVA can generate sequences for diverse and complex backbones independent of structure prediction confidence. desHE2, a *de novo* asymmetric β-propeller, was solved to 1.48Å with the backbone closely matching the designed model (Cα-RMSD: 0.80Å, PDB: 12WO, Fig. 2F). The designed zinc was clearly resolved in the electron density and the metal positioned within 0.90Å of the designed location. desE2D, a *de novo* half-open TIM barrel, diffracted at 1.55Å and showed excellent backbone agreement with the design (Cα-RMSD: 1.42Å, PDB: 12WN, Fig. 2G), with deviations localized to a less ordered C-terminus. Likewise, a crystal structure of desDEH further confirms backbone fidelity (Cα-RMSD: 0.74Å, PDB: 12WM, Fig. S10A). In the latter two cases, the metal site was ambiguous in the electron density. However, XAS confirms the presence of zinc; the Fourier transform of EXAFS shows shorter first-shell distance to free ZnCl₂, confirming protein-bound metal (Fig. 2H, Fig. S10B), with ICP-MS indicating 1:1 stoichiometry (Fig. 3C). The EXAFS beat patterns show lower intensities than the desH2C2 design (Fig. 2I), suggesting ligation by lighter atoms (nitrogen or oxygen) consistent with the designed atom composition (Table S4, Fig. S10C).

As a negative control to probe the boundaries of dEVA’s design capabilities, we examine desD1 where the chosen design is far from the knee point (Fig. 3A–B). The designed site contains only a single ligand within binding distance—a coordination environment that is chemically insufficient for stable metal chelation. Experimentally, there is no detectable binding; competition titration against MagFura-2 showed no apparent binding affinity and ICP-MS confirmed no zinc association (Fig. 3C–D).

## dEVA is sensitive to the models’ training data composition.

Despite our designs showing experimental success and consistency with the designs, we expected dEVA to create high affinity sites because of its additional consideration of the second-shell environment. However, our results show that most of the designs have weaker affinity than characterized natural metalloproteins, suggests that something was not being adequately captured during the design protocol. Post-hoc, we predicted the structure of our negative control design desD1. Despite excellent overall structural agreement with the design, AlphaFold-3^24^ assigns high-confidence to the predicted metal (Zn pLDDT: 86.55) and positions it in the same place as the low-confidence design (Fig 3A). By standard computational metrics and filtering criteria, this would have been classified as a success; this ambiguity prompted us to evaluate what these models learned by examining their training data.

We began with the structural training datasets of both LigandMPNN and Metal3D—the two models used for dEVA metalloprotein design—and analyzed the coordination environments within 3.0Å of all deposited zincs in the first biological assembly (Fig. S11–S12). Across both datasets, we found that approximately 10% of zinc sites had zero coordinating ligands, and over 54% had two or fewer residues (Fig. 3E). The distribution of the atom compositions in these sites reflects this heterogeneity; the most prevalent coordination pattern is a single, monodentate oxygen with three unoccupied coordination sites, while well-defined tetrahedral environments and catalytic sites are comparatively rare (Fig. 3F). Closer inspection of the structures reveals that many of the poorly coordinated sites may reflect crystallization additives or soaking agents rather than biologically relevant co-factors. Zinc ions exhibit non-specific surface binding, particularly near aspartates, glutamates, and histidines^25^. As a result, some depositions report over 20 resolved zinc atoms in their electron density (e.g. PDB: 2EJC_A), while others contain only a few zinc atoms tightly associated with just one ligand (e.g. PDB: 3IVB_A) (Fig. S13). These artefacts explain the disproportionate prevalence of carboxylate-mediated zincs in deep learning training datasets and the resulting confident predictions.

To address this directly, we trained Metal3D-Clean and excluded all zinc sites with fewer than three coordinating residues within 3.0Å. Comparing Metal3D and Metal3D-Clean on the same held-out test set reveals a clear and expected tradeoff: Metal3D-Clean assigns substantially lower predicted probabilities and recall values for poorly coordinated sites (0–2 residues) while preserving performance on well-coordinated sites (Fig. 3G). As model behavior is directly shaped by training data composition, dEVA’s designs are inherently sensitive to the data its underlying models are trained on.

## The design of a *de novo* bi-zinc metalloenzyme.

We next sought to exploit this sensitivity by tailoring the objectives for dEVA to enable the zero-shot design of a functional metalloenzyme. dEVA therefore needed a model that approximates *p(catalytic metal | sequence, structure)*. This compelled us to train Metal3D-Cat on only annotated, catalytic zincs from MAHOMES-II^26^ to discriminate the first- and second-shell chemical environments that govern zinc-mediated catalysis^3,27^ (see **Methods**, Fig. 3H).

Analysis of the training data indicates that over half of the annotated zinc sites belong to hydrolases; of these, one-third catalyze the hydrolysis of phosphate-oxygen bonds (Fig. 3I). Notably, while most hydrolytic active sites employ a single catalytic zinc, a substantial fraction of zinc-containing enzymes use a bi- or tri-nuclear zinc center. In mono-nuclear zinc hydrolases, zinc acts as a Lewis acid to activate a water; in binuclear-zinc sites, the two metal centers play complementary roles in activating the nucleophilic hydroxide, orienting the substrate, and stabilizing the charge in the transition state^28,29^ (Fig. 3J).

**Figure 4.**
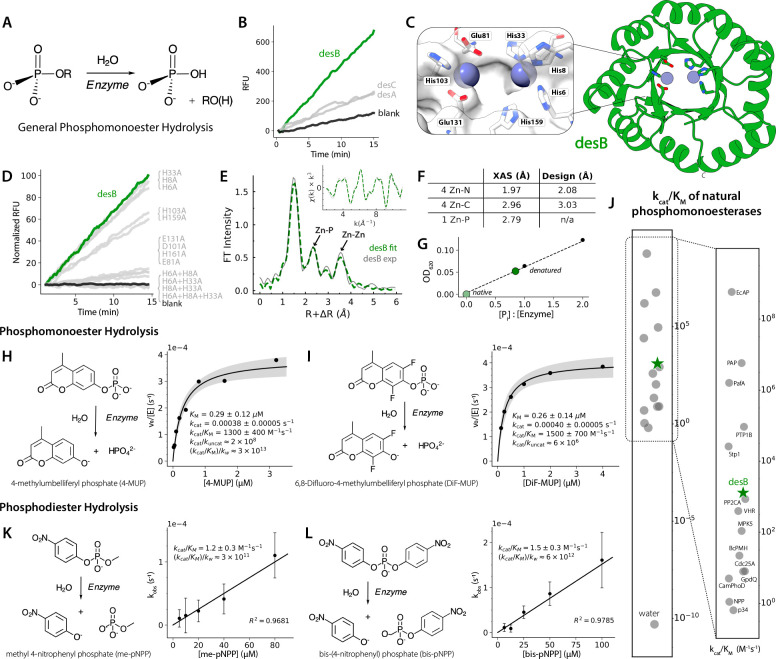
Zero-shot design of a de novo binuclear zinc metalloenzyme. (A) General mechanism of phosphomonoester hydrolysis (B) Initial fluorescence-based screen of desA, desB, and desC against 4-MUP; desB (green) shows the highest activity. (C) Cartoon representation of the dEVA design model for desB, a *de novo* TIM barrel harboring a bi-zinc active site (blue spheres). (D) Screen of the alanine mutants of first- and second-shell active site residues confirms catalytic dependence on the designed bi-zinc site. (E) Zn K-edge EXAFS of desB. Structural analysis of the bi-zinc active site; experimental (black) and fitted (green) spectra fit with a Zn-Zn scattering path at 4.3Å (see Table S4). (F) Comparison of EXAFS distances and design model. (G) Inhibition of phosphomonesterase activity by orthophosphate. Data (n=1) fit to a hyperbolic inhibition model; K_i,app_ corrected for substrate competition. (H, I) Michaelis-Menten kinetics of desB for phosphomonoester substrates 4-MUP and DiF-MUP. Shaded region represents ±1 standard error. Measurements were performed in biological triplicate. (J) Comparison of desB catalytic efficiency (k_cat_/K_M_) against literature values for natural phosphomonoesterases. (K, L) Linear kinetics of desB for me-pNPP and bis-pNPP. Measurements were performed in biological duplicate.

To design a *de novo* metalloenzyme, we began by constructing a library of unconditionally generated backbones with Protpardelle and filtered to a subset of TIM-barrel scaffolds—a fold whose functional plasticity has made it abundant across all six enzyme classes^30^. For each scaffold, an initial full sequence was designed with ensemble Caliby^31^, a Potts-based sequence design model that conditions on synthetic structural ensembles to remove non-structural sequence bias. Then, dEVA was employed to explore catalytic zinc configurations within the barrel cavity. dEVA identified only three Pareto-optimal solutions; these three designs (Fig. S14)—desA, desB, and desC—were individually screened for hydrolytic activity against phospho-ester substrates (Fig. 4A), reflecting the chemical reaction most represented in the Metal3D-Cat training data.

Of the three candidates, desB emerged as the most active, catalyzing phosphomonoester hydrolysis with a rate significantly above background (Fig 4B). This is particularly notable as desB harbors a bi-nuclear zinc active site (Fig. 4C)—the same cooperative two-metal mechanism that underlies the exceptional efficiency of many naturally occurring metallohydrolases^29^. We chose to characterize this design in full.

desB is well-folded and monomeric, exhibiting high thermostability as assessed by SEC and CD, respectively (Fig. S15–S16A). The binuclear zinc site of desB is an entirely *de novo* motif; motif searches against the PDB using both Folddisco^32^ and RCSB Structure Motif Search^33^ returned no structural motif neighbors, and BLAST^34^ returned no sequence homologs. The novelty of this active site is further supported by the absence of bi-zinc sites among structural neighbors identified by Foldseek^35^.

To verify that the designed zinc site drives catalysis, we confirmed that EDTA chelation significantly reduces activity (Fig. S16B). However, metal chelation also destabilizes the protein (Fig. S16C), precluding determination of zinc binding affinity. Alanine scanning mutagenesis of the first-and second-shell residues showed that mutations to the designed site affect activity (Fig. 4D, Fig. S17). Metal stoichiometry was independently determined by ICP-MS, confirming that desB coordinates two zinc ions per monomer (Table S7). pH profiling revealed optimal activity under alkaline conditions (Figs. S18), indicative of a binuclear zinc site in which two proximal zinc ions lower the pKa of metal-bound water to ~7, thereby generating a hydroxide nucleophile for attack at alkaline pH^28,29^ (Fig. 3J).

To directly characterize how these zinc ions are organized in solution, we performed XAS. desB showed a high X-ray fluorescence count that is consistent with two zinc ions. Notably, the Zn-Zn distance detected in solution (~4.3Å) is shorter than the 5.8Å separation in the design model yet falls within the range of observed catalytically competent binuclear zinc hydrolases^36^. Fitting the Zn K-edge EXAFS yielded scattering paths consistent with 4 Zn-N, 1 Zn-P, and 1 Zn-Zn interactions (Fig. 4E–F, Fig. S19). The prominent Zn-P contribution indicates direct coordination of phosphate to the metal center and suggests a closely associated, bridged configuration upon binding. Phosphate coordination was independently confirmed by a malachite green assay (Fig. 4G, Fig. S20), with bound phosphate detected only upon denaturation. Phosphate itself inhibits desB activity with a K_i,app_ of 1.0±0.2μM, consistent with coordination at a binuclear zinc active site and comparable to the inhibition constants reported for natural alkaline phosphatases^37^ (Fig. S21).

The hydrolysis of phosphomonoesters is among the most energetically demanding reactions in biology, with uncatalyzed half-lives >500,000 years^38^. desB overcomes this barrier with catalytic efficiencies (k_cat_/K_M_) of 1300±400 and 1500±700 M^−1^s^−1^ for 4-MUP and DiF-MUP, respectively (Fig. 4H–I). Compared to literature values, desB activity is within the range of natural phosphomonoesterases (Fig. 4J, Table S8). Its rate enhancement ((k_cat_/K_M_)/k_w_) is 3×10^13^ for 4-MUP, the highest reported rate enhancement for any *de novo* designed hydrolase to date^16^. desB continually turns over substrate, as confirmed by product accumulation over hours at sub-stoichiometric enzyme concentrations (Fig. S22).

Remarkably, desB also catalyzes the hydrolysis of phosphodiesters, a chemically more demanding reaction class (with uncatalyzed half-lives >13 million years^38^) distinguished by a different charge state and transition state geometry. desB hydrolyzes both me-pNPP and bis-pNPP with a rate enhancement of 3×10^11^ and 6×10^12^, respectively (Fig. 4K–L). Their k_cat_/K_M_ of 1.2±0.3 and 1.5±0.3 M^−1^s^−1^ makes desB also comparable to natural enzymes with characterized phosphodiesterase activity (Fig. S23, Table S9). This substrate promiscuity suggests that the catalytic efficiency is not limited by the size of the diester moiety, consistent with the original design of an exposed active site.

## Discussion.

The design of *de novo* enzymes represents one of the most demanding tests of deep learning methods. While previous enzyme designs have relied on scaffolding known catalytic motifs, this constrains solutions to geometries that evolution has already conceived. We demonstrate that this constraint is not necessary; by starting from no natural template, no theozyme geometry, and no evolutionary data, dEVA yielded a *de novo* metalloenzyme in zero-shot. The resulting design has no structural precedent and catalytic efficiency comparable to natural phosphatases—catalyzing two of the most energetically demanding reactions in biology.

This work draws inspiration from how natural selection forged highly proficient function. In nature, the evolution of enzymes is widely thought to have begun with “generalist” catalysts that, under millennia of selective pressure, became highly efficient and substrate-specific machines^39^. Analogously, the design of *de novo* functional proteins may begin with the design of promiscuous catalysts as a bed-ground for future protein engineering efforts. desB is one such catalyst: its active site sits in a non-specific open cavity, and its promiscuity for model phosphomonoester and phosphodiester substrates is unpaired compared to the promiscuity of natural enzymes^40^. This opens the door for future engineering of desB to tune its specificity and tailor its catalysis.

To navigate sequence space, dEVA similarly draws from evolutionary biology. Natural selection explores sequence space through iterative mutation and selection, implicitly sampling epistatic interactions. By leveraging a genetic algorithm, dEVA enables exploration of these interdependencies—though its success ultimately rests on both the quality of the training data and sensitivity of the models used for each defined objective^41^. Our findings here represent a cautionary tale on training deep learning models without consideration of the physiochemical reality of macromolecules. Continued development of models that learn the biophysical and biochemical determinants of protein function will enable better formulation of the design objectives necessary for efficient catalysis. dEVA offers a starting point for balancing these objectives and we envision as a flexible platform for functional protein design. Ultimately, the design of diverse enzymes will benefit from accessing other catalytic solutions yet to be conceived by natural evolution.

## Supplementary Material

Supplement 1

## Data Availability

All data are available in the main text or as supplementary materials, including all reference literature values, ordered protein sequences and mutants, and design models. Protein crystal structure coordinates and structure factors are available in the Protein Data Bank with PDB IDs 12WM, 12WN, and 12WO.

## Code availability.

dEVA and all associated model weights are available as a modular framework via GitHub at https://github.com/ProteinDesignLab/dEVA.
